# Dynamic Fractional Flow Reserve from 4D-CTA: A Novel Framework for Non-Invasive Coronary Assessment

**DOI:** 10.3390/jimaging11100330

**Published:** 2025-09-24

**Authors:** Shuo Wang, Rong Liu, Li Zhang

**Affiliations:** 1 Key Laboratory of Particle and Radiation Imaging, Department of Engineering Physics, Ministry of Education, Tsinghua University, Beijing 100084, China; shuo-wan19@mails.tsinghua.edu.cn; 2 Coronary Heart Disease Center, National Center for Cardiovascular Disease and Fuwai Hospital, Chinese Academy of Medical Sciences and Peking Union Medical College, Beijing 100037, China; liurongwjt@sina.com

**Keywords:** fractional flow reserve, 4D-CTA, dynamic hemodynamics, patient-specific modeling

## Abstract

Current fractional flow reserve computed tomography (FFR_CT_) methods use static imaging, potentially missing critical hemodynamic changes during the cardiac cycle. We developed a novel dynamic FFR_CT_ framework using 4D-CTA data to capture temporal coronary dynamics throughout the complete cardiac cycle. Our automated pipeline integrates 4D-CTA processing, temporally weighted geometric modeling, and patient-specific boundary conditions derived from actual flow measurements. Preliminary validation in three patients (four vessels) showed that dynamic FFRCT values (0.720, 0.797, 0.811, and 0.952) closely matched invasive FFR measurements (0.70, 0.78, 0.78, and 0.94) with improved accuracy compared to conventional static methods. The dynamic approach successfully captured physiologically relevant hemodynamic variations, addressing inter-patient variability limitations of standardized approaches. This study establishes the clinical feasibility of dynamic FFR_CT_ computation, potentially improving non-invasive coronary stenosis assessment for clinical decision-making and treatment planning.

## 1. Introduction

Cardiovascular diseases (CVDs) remain the leading cause of mortality worldwide, with coronary artery disease (CAD) representing the predominant contributor to this global health burden [1,2]. CAD is characterized by atherosclerotic plaque accumulation leading to arterial stenosis, which can result in myocardial ischemia and infarction [3,4]. Despite significant advances in diagnostic approaches, accurately assessing coronary stenosis severity and its functional significance remains a critical challenge.

Coronary artery hemodynamics has become essential for understanding cardiovascular physiology and pathology [5]. Computational approaches, particularly computational fluid dynamics (CFD), provide robust frameworks for modeling complex blood flow patterns and investigating key hemodynamic parameters in vascular geometries [6,7].

The fractional flow reserve (FFR) has been established as the gold-standard metric for functionally assessing the severity of coronary artery stenosis, defined as the ratio of pressure downstream of the stenosis to aortic pressure [8]. Traditional FFR measurement requires invasive catheterization with pressure wire insertion, which poses inherent risks to patients and incurs substantial healthcare costs [9]. This limitation has driven the development of non-invasive computational methodologies for FFR prediction, leading to significant advances in virtual FFR (vFFR) derived from coronary computed tomography angiography (CCTA) [10,11]. Additionally, the Quantitative Flow Ratio (QFR), which utilizes angiographic data combined with CFD principles, has demonstrated high accuracy and efficiency in diagnosing ischemia-causing lesions [12]. These computational advancements represent a paradigm shift toward reducing reliance on invasive procedures while maintaining diagnostic precision.

Coronary computed tomography angiography (CCTA) has evolved into a robust imaging technique for the detection, quantification, and characterization of coronary atherosclerosis [13]. The technique’s high negative predictive value makes it particularly valuable for conclusively excluding CAD in patients with a low pretest probability of disease [14]. Recent technological advances in CCTA have enhanced both spatial and temporal resolution, enabling motion-free imaging of coronary arteries and facilitating the identification of high-risk atherosclerotic plaque features [15]. Furthermore, FFR derived from CCTA (FFR_CT_) has emerged as a promising non-invasive tool for evaluating the physiological significance of coronary artery stenosis, bridging the gap between anatomical and functional assessment [16].

The development of sophisticated open-source software platforms has significantly accelerated progress in cardiovascular CFD simulations, addressing both cost and accessibility concerns in the research community [17]. Notable platforms include SimVascular and CRIMSON, which provide comprehensive pipelines for image-based vascular modeling, mesh generation, and blood flow simulation [6,7]. Among these tools, OpenFOAM has gained significant traction as a versatile and widely adopted open-source CFD framework, offering extensive solvers, robust computational capabilities, and customizable workflows suitable for complex cardiovascular hemodynamics problems [18,19,20].

Despite these technological advances, the application of computational methodologies to coronary artery hemodynamics faces several persistent challenges. Current approaches often require significant manual effort for preprocessing, boundary condition setup, and solver configuration, limiting their clinical translation and widespread adoption [21]. Moreover, the computational complexity and time requirements of traditional CFD simulations present barriers to real-time clinical implementation [22]. A fundamental limitation of existing methods lies in their reliance on empirically derived parameters and standardized boundary conditions, including uniform outlet pressures, generalized inlet/outlet flow profiles, and homogeneous material properties for vessel walls and uniform fluid properties for blood flow. This one-size-fits-all approach fails to adequately capture the substantial inter-patient anatomical and physiological variability, leading to systematic errors in FFR_CT_ calculations and compromised diagnostic accuracy. The heterogeneity in coronary anatomy, microvascular resistance, and hemodynamic characteristics across different patient populations necessitates the development of personalized computational frameworks that can dynamically adapt boundary conditions and material properties to individual patient physiology, rather than applying universal assumptions that may not reflect the complex pathophysiological reality of coronary artery disease.

To address these limitations, 4D-CTA represents a significant advancement in cardiovascular imaging, enabling dynamic assessment of coronary hemodynamics throughout the cardiac cycle [23]. Unlike static imaging, 4D-CTA provides temporal resolution to capture pulsatile flow patterns and dynamic vessel geometry changes [24], creating opportunities for more accurate coronary stenosis evaluation through dynamic FFR computation that accounts for temporal hemodynamic variations.

However, current methods have not fully exploited 4D-CTA’s temporal capabilities for dynamic FFR_CT_ computation. Integrating 4D-CTA data with computational hemodynamic modeling presents unique challenges requiring sophisticated frameworks capable of handling time-varying geometries while maintaining clinical computational efficiency. Validation of dynamic FFR_CT_ methodologies against clinical standards remains limited.

This study introduces a novel computational approach for dynamic FFR_CT_ calculation based on 4D-CTA imaging data. The primary objective is to develop and validate a comprehensive methodology leveraging 4D-CTA temporal information to compute dynamic FFR values throughout the cardiac cycle, providing more physiologically relevant coronary stenosis assessment. The approach integrates advanced image processing, patient-specific geometric modeling, and efficient computational algorithms for practical clinical implementation.

This research advances personalized cardiovascular medicine by providing clinicians with dynamic, patient-specific hemodynamic information to improve diagnostic accuracy and treatment planning, ultimately contributing to enhanced clinical decision-making.

## 2. Materials and Methods

**Figure 1:**
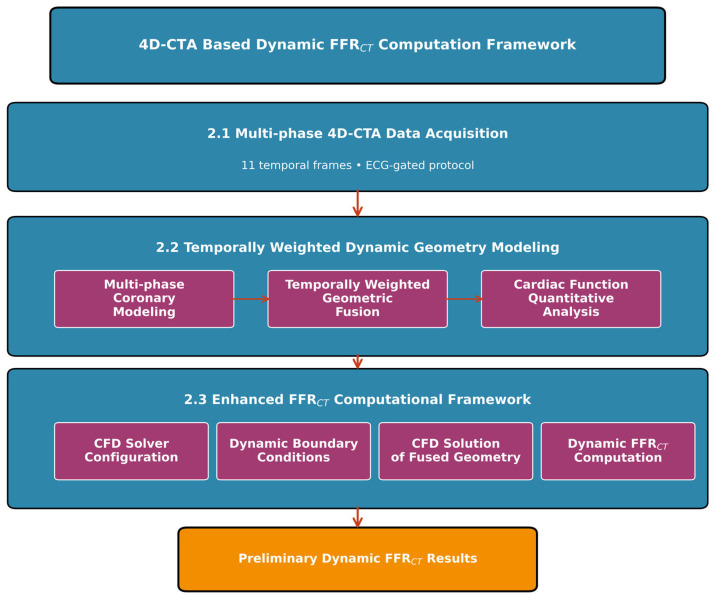
Schematic overview of the 4D-CTA-based dynamic FFR_CT_ computation framework.

This study establishes a 4D-CTA-based dynamic FFR_CT_ computation methodology to explore the feasibility of incorporating temporal cardiac dynamics into virtual FFR assessment (Figure 1). The framework comprises three core components: first, the acquisition of multi-phase 4D-CTA data covering the complete cardiac cycle to capture dynamic coronary geometry; second, the development of a temporally weighted dynamic coronary geometry modeling algorithm that integrates multi-phase dynamic modeling, temporally weighted geometric fusion, and quantitative analysis of cardiac function to create hemodynamically informed vascular geometric representations; third, the implementation of an enhanced FFR_CT_ computational framework that combines dynamic geometric characterization with physiologically guided boundary conditions. This preliminary approach addresses the limitations of conventional static geometric modeling by capturing dynamic vessel characteristics throughout the cardiac cycle, potentially improving the clinical relevance of FFR_CT_ calculations, though further validation with larger datasets is needed to establish clinical utility.

### 2.1. Patient Data Acquisition

Patients with suspected coronary artery disease scheduled for coronary angiography examination were enrolled in this study following comprehensive clinical evaluation and cardiovascular risk assessment. Inclusion criteria included (1) age 18–80 years, (2) clinical suspicion of coronary artery disease requiring coronary assessment, (3) sinus rhythm with heart rate variability <10%, (4) ability to provide informed consent and cooperate with examination procedures, and (5) at least one major coronary artery with moderate stenosis (30–90% diameter stenosis). Exclusion criteria included (1) previous coronary artery bypass surgery or percutaneous coronary intervention, (2) severe arrhythmias (atrial fibrillation or frequent ventricular ectopy), (3) severe heart failure (LVEF < 30%), (4) severe renal dysfunction (eGFR < 30 mL/min/1.73 m^2^), (5) contrast allergy, (6) pregnancy or lactation, (7) poor 4D-CTA image quality preventing reliable geometric reconstruction, and (8) complete coronary occlusion (100% stenosis).

Four-dimensional coronary computed tomography angiography was performed using a SOMATOM Force dual-source CT scanner (Siemens Healthineers, Erlangen, Germany) with optimized cardiac imaging parameters. The imaging protocol was specifically designed for dynamic coronary artery assessment with a slice thickness of 0.75 mm and reconstruction algorithms optimized for vascular imaging. The 4D-CTA protocol generated 11 temporal frames covering the complete cardiac cycle from 0 to 100% of the R-R interval, with each frame representing approximately 9% of the cardiac cycle’s duration. This temporal sampling strategy enabled comprehensive visualization of coronary artery dynamics throughout both the systolic and diastolic phases, capturing subtle changes in luminal dimensions and wall motion characteristics. The dual-source configuration achieved a high temporal resolution of approximately 75–83 milliseconds per frame through simultaneous data acquisition from two X-ray sources positioned at 90-degree angles, effectively minimizing motion artifacts even in patients with elevated heart rates. The 11-frame reconstruction provided sufficient temporal density to analyze coronary flow dynamics, identify optimal cardiac phases for stenosis assessment, and evaluate functional parameters including coronary artery distensibility and phasic flow variations throughout the cardiac cycle.

Prospective ECG triggering with continuous cardiac rhythm monitoring ensured accurate temporal registration across all 11 cardiac phases. Non-ionic iodinated contrast medium (350 mgI/mL, 80–100 mL) was administered at 5.0–6.0 mL/s with the bolus tracking technique, triggering acquisition when ascending aortic attenuation reached 100 Hounsfield units above baseline. Patient demographics, vital signs including heart rate and blood pressure, and cardiac functional indices were systematically recorded. Heart rate stability and rhythm regularity were continuously monitored throughout the examination to ensure optimal 4D reconstruction quality. Image quality was assessed using a 5-point scale for motion artifacts, with only studies scoring ≤2 included in the analysis. All patients provided written informed consent, and the study was approved by the institutional ethics committee in accordance with the Declaration of Helsinki.

### 2.2. Temporally Weighted Dynamic Coronary Geometry Modeling

The temporally weighted dynamic coronary geometry modeling from 11-frame 4D-CTA data represents a paradigm shift from conventional static modeling approaches to hemodynamically informed geometric representation. Traditional coronary artery modeling typically relies on single-phase imaging, most commonly end-diastolic CTA acquisitions, that fails to capture the dynamic nature of coronary vessels throughout the cardiac cycle, and consequently introduces systematic errors in subsequent computational fluid dynamics analyses.

#### 2.2.1. Dynamic Multi-Phase Coronary Modeling

**Figure 2:**
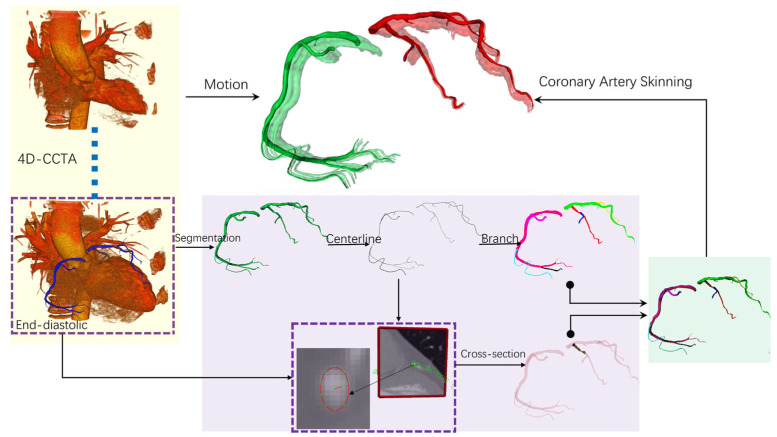
Comprehensive workflow for dynamic coronary artery model generation.

To achieve dynamic coronary modeling, we construct temporally consistent vascular geometric models across 11 temporal phases covering the complete cardiac cycle. Building upon our established methodology in coronary dynamic sequence generation and geometric deformation [25], this section focuses on creating a comprehensive dynamic coronary model that serves as foundational data for the subsequent temporally weighted geometric fusion algorithm. Our methodology for generating dynamic coronary artery models follows a comprehensive workflow, as illustrated in Figure 2. Beginning with 4D-CCTA data acquisition, we extract end-diastolic coronary images as the foundation for subsequent processing. The workflow proceeds through coronary arterial tree construction, which involves vessel segmentation, centerline extraction, and branch identification from the end-diastolic reference frame. The critical innovation lies in our coronary artery skinning methodology, which transforms the static end-diastolic model into a comprehensive multi-phase representation through specialized deformation techniques. Cross-sectional analysis reveals the dynamic geometric parameters that undergo temporal variation throughout the cardiac cycle. The final motion simulation generates temporally consistent vascular models with distinct visualization for different coronary branches, providing the foundational geometric data for the subsequent temporally weighted fusion algorithm. This integrated approach creates clinically valuable dynamic coronary models that serve as inputs for hemodynamically informed geometric fusion and computational fluid dynamics analysis.

Our approach begins with coronary artery segmentation performed on end-diastolic CTA images, where vessels are maximally dilated with optimal contrast enhancement and minimal motion artifacts. The segmentation employs an enhanced multi-scale Frangi vesselness filter [26], where the vesselness measure $V_{0}\left(s\right)$ at a given scale σ follows the established framework. The final vesselness measure is obtained by computing the maximum response over a range of scales: $V=\underset{\sigma_{\min}<\sigma <\sigma_{\max}}{\max}V_{0}\left(s\right)$, where $\sigma_{min}=0.5$ mm and $\sigma_{max}=2.5$ mm for coronary arteries.

Following the initial segmentation, we extract the vessel centerline using a fast-marching algorithm combined with backtracking, represented as a series of points $P_{i}\left(x_{i},y_{i},z_{i}\right)$ with associated vessel radius information. The arterial tree topology is analyzed using a hierarchical approach, with branch points characterized by bifurcation angles and cross-sectional area ratios following a modified version of Murray’s law for coronary arteries [27] as follows: $r_{p}^{2.6}=r_{d1}^{2.6}+r_{d2}^{2.6}$, where $r_{p}$ represents the radius of the parent vessel, and $r_{d1}$ and $r_{d2}$ represent the radii of the two daughter branches, respectively.

The critical innovation lies in our dynamic sequence generation methodology, which transforms the static end-diastolic model into a comprehensive multi-phase representation. The cardiac cycle is temporally sampled into discrete phases, with centerline evolution described by the time-dependent curve $C\left(s,t\right)=\left(x\left(s,t\right),y\left(s,t\right),z\left(s,t\right)\right)$, where s represents the arc length parameter and t denotes the cardiac phase. The vessel cross-sections undergo simultaneous deformation, modeled as ellipses with time-varying parameters following volume conservation as follows: $\pi a\left(t\right)b\left(t\right)=A_{0}\left(1+\epsilon_{r}\left(t\right)\right)$, where $a\left(t\right)$ and $b\left(t\right)$ represent the time-varying semi-major and semi-minor axes of the elliptical cross-section, respectively; $A_{0}$ denotes the reference cross-sectional area at the baseline state; and $\epsilon_{r}\left(t\right)$ represents radial strain within the physiological range of ±5%.

Inter-phase geometric correspondence is established through our coronary artery skinning weight calculation methodology. The deformation process employs the following biharmonic energy minimization: $\underset{W}{\min}\mathrm{tr}\left(W^{T}LW\right)$ subject to boundary conditions BW=D, where W represents the weight matrix, L is the discrete bi-Laplacian operator, B denotes the boundary constraint matrix, D represents the boundary condition vector, and the constraints encode anatomical boundary conditions. Once the normalized skinning weights $w_{i,j}^{\prime }$ are computed, vessel wall deformation is achieved through weighted linear blend skinning as follows:

(1) $$v_{i}^{\prime }=\Sigma_{j=1}^{N_{c}}w_{i,j}^{\prime }T_{j}v_{i}$$

where $v_{i}^{\prime }$ is the deformed vertex position, $T_{j}$ is the rigid body transformation matrix of centerline segment j, and $N_{c}$ is the total number of segments. This ensures smooth and physiologically plausible vessel wall deformation throughout the cardiac cycle while maintaining structural integrity.

The dynamic modeling incorporates rigorous anatomical and mechanical constraints with temporal coherence enforced through position constraints between consecutive phases, ensuring smooth motion transitions and periodic boundary conditions. The final multi-phase output comprises 11 complete geometric models $G_{1},G_{2},\ldots ,G_{11}$, each containing phase-specific centerlines $C_{i}\left(s\right)$; vessel surface meshes $M_{i}$ with deformed vertex positions $v_{i}^{\prime }$; and dynamic geometric parameters including time-varying cross-sectional areas $A_{i}\left(s\right)$, vessel radii $r_{i}\left(s\right)$, and local curvatures $\kappa_{i}\left(s\right)$. This approach provides reliable input data for the subsequent temporally weighted geometric fusion algorithm.

#### 2.2.2. Temporally Weighted Geometric Fusion Algorithm

The core contribution of our approach lies in the development of a temporally weighted geometric fusion algorithm that addresses the limitations of static geometric modeling in hemodynamic computations. Rather than selecting a single representative phase or performing simple geometric averaging, our algorithm creates a hemodynamically informed geometric representation that better reflects the flow environment experienced by blood throughout the cardiac cycle.

**Figure 3:**
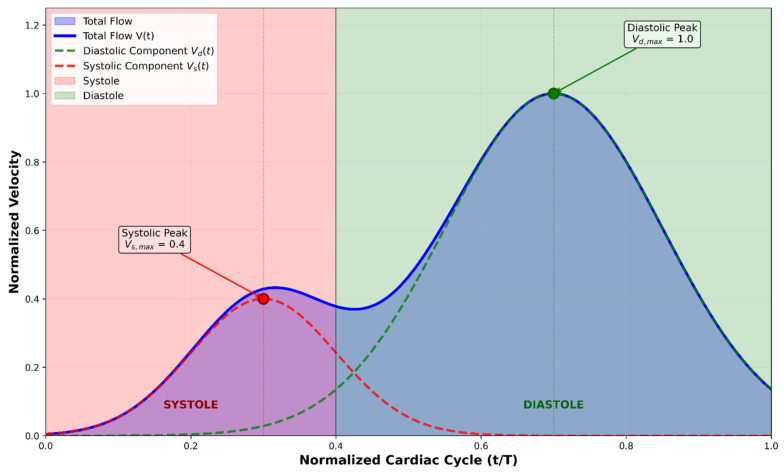
Coronary artery inlet flow velocity profile.

Prior to temporal fusion, we establish the hemodynamic foundation through coronary inlet flow velocity characterization. Coronary flow exhibits a distinctive biphasic pattern with diastolic dominance, fundamentally differing from systemic circulation. In the absence of patient-specific measurements, we employ a physiologically based empirical model consistent with established computational hemodynamics practices [28]. As shown in Figure 3, the coronary flow velocity profile V(t) represents the superposition of diastolic $V_{d}\left(t\right)$ and systolic $V_{s}\left(t\right)$ components. The diastolic component dominates with peak velocity $V_{d,max}=1.0$ at t/T=0.7, while the systolic component shows reduced peak velocity $V_{s,max}=0.4$ at t/T=0.3, reflecting the characteristic diastolic-dominant pattern.

The temporal velocity profile is constructed by decomposing the cardiac cycle into constituent phases. Given cardiac period T=60/HR where HR represents heart rate in beats per minute, the coronary flow velocity becomes

(2) $$V\left(t\right)=V_{d}\left(t\right)+V_{s}\left(t\right)$$

Based on reduced intramyocardial compression during ventricular relaxation [29], the components are modeled as

(3) $$V_{d}\left(t\right)=V_{d,\max}\cdot \exp\left(-{\left(t/T-0.7\right)}^{2}/2\sigma_{d}^{2}\right)$$

(4) $$V_{s}\left(t\right)=V_{s,\max}\cdot \exp\left(-{\left(t/T-0.3\right)}^{2}/2\sigma_{s}^{2}\right)$$

where $\sigma_{d}=0.15$ and $\sigma_{s}=0.1$ control phase durations, with $V_{d,\max}>V_{s,\max}$ ensuring diastolic dominance [30].

In clinical physiological assessments, alterations in the resting phasic coronary flow velocity profile serve as indicators of coronary pathophysiology. The diastolic-to-systolic velocity ratio (DSVR) reflects the coronary microcirculatory wave patterns that distinguish coronary circulation from peripheral vascular beds [11], making accurate flow profile characterization essential for pathological evaluation. Prior to temporal fusion, patient-specific flow velocity profile computation personalizes the established hemodynamic foundation using 4D-CTA temporal data. Building upon the physiologically based empirical model $V\left(t\right)=V_{d}\left(t\right)+V_{s}\left(t\right)$ that captures the characteristic coronary flow dynamics, patient-specific calibration is achieved through temporal contrast enhancement analysis from 4D-CTA acquisition. The normalized contrast enhancement ratio $R\left(t_{i}\right)=\frac{HU\left(t_{i}\right)-{HU}_{base}}{{HU}_{peak}-{HU}_{base}}$ extracted from the coronary inlet region serves as a patient-specific scaling factor that modulates the baseline flow profile (${HU}_{base}$: pre-contrast baseline; ${HU}_{peak}$: maximum contrast enhancement). The personalized velocity profile becomes $V_{p}\left(t_{i}\right)=\alpha \cdot R\left(t_{i}\right)\cdot V\left(t_{i}\right)$, where α represents a patient-specific calibration factor derived from cardiac output estimation and vessel geometry analysis from 4D-CTA morphological data [31], and $V\left(t_{i}\right)$ denotes the baseline physiological profile at temporal phase i. This approach ensures that the resulting velocity profile maintains the characteristic biphasic coronary flow pattern while incorporating individual patient hemodynamic variations extracted from 4D-CTA, serving as personalized inlet boundary conditions for subsequent temporal fusion weighting.

The temporally weighted geometric fusion algorithm processes the 11-frame temporal sequence to extract phase-specific geometric parameters including centerline coordinates $C_{i}\left(s\right)=\left[x_{i}\left(s\right),y_{i}\left(s\right),z_{i}\left(s\right)\right]$, cross-sectional areas $A_{i}\left(s\right)$, local curvatures $\kappa_{i}\left(s\right)=\left|dT/ds\right|$, and torsion $\tau_{i}\left(s\right)$ for each temporal phase i.

The hydraulic diameter is defined as $D_{h,i}\left(s\right)=2\sqrt{A_{i}\left(s\right)/\pi }$, providing a characteristic length scale for non-circular cross-sections at each cardiac phase [32]. The reference area $A_{ref}\left(s\right)=\underset{j}{\max}A_{j}\left(s\right)$ corresponds to the maximum cross-sectional area across all cardiac phases at each spatial location. The local Reynolds number $Re_{i}\left(s\right)=\rho {\bar{v}}_{i}D_{h,i}\left(s\right)/\mu$ is computed using the average velocity ${\bar{v}}_{i}\left(s\right)=Q_{i}\left(s\right)/A_{i}\left(s\right)$, where the reference Reynolds number ${Re}_{ref}=500$ represents typical coronary flow conditions [33].

**Figure 4:**
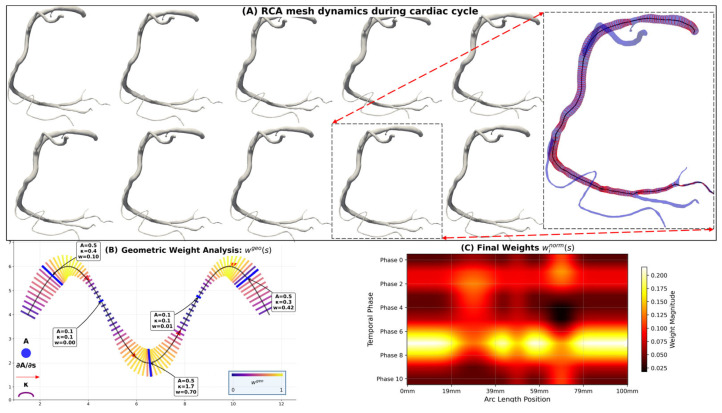
Temporally weighted geometric fusion algorithm for RCA mesh dynamics. (**A**) Sequential RCA mesh configurations showing vessel geometry variations throughout the cardiac cycle. The highlighted blue mesh shows red contours representing vessel cross-sections, with the black lines showing centerlines. (**B**) Geometric parameter analysis showing centerline coordinates, cross-sectional areas, curvatures, and torsion variations along the RCA arc length. (**C**) Final normalized weights heatmap showing temporal–spatial weight distribution across cardiac phases and vessel arc length, where higher weights indicate greater hemodynamic significance for the temporal fusion algorithm.

The temporal fusion employs a comprehensive weighting strategy that incorporates multiple geometric indicators correlating with hemodynamic significance. As illustrated in Figure 4A, the right coronary artery (RCA) mesh configurations vary significantly across cardiac phases, necessitating sophisticated weighting to capture hemodynamic relevance. The weighting considers Reynolds number variations, Dean flow phenomena, and stenotic flow acceleration across different cardiac phases as follows:

(5) $$w_{i}\left(s\right)=w_{i}^{base}\cdot w_{i}^{geo}\left(s\right)$$

The base weight provides global normalization

(6) $$w_{i}^{base}=\frac{\left|V_{p}\left(t_{i}\right)\right|}{\sum_{j=0}^{10}\left|V_{p}\left(t_{j}\right)\right|}$$

where $V_{p}\left(t_{i}\right)$ represents the inlet flow velocity sampled from the coronary flow profile at the i-th temporal phase, and the base weight provides global normalization across all cardiac phases.

The geometric complexity weight captures local hemodynamic significance (Figure 4B) as follows:

(7) $$\begin{array}{c} w_{i}^{geo}\left(s\right)=\alpha \cdot \frac{\left|dA_{i}/ds\right|}{A_{i}\left(s\right)}\cdot \frac{Re_{i}\left(s\right)}{{Re}_{ref}}+\beta \cdot \frac{\kappa_{i}\left(s\right)\cdot D_{h,i}\left(s\right)}{2}\cdot \sqrt{\frac{Re_{i}\left(s\right)}{Re_{ref}}}\\ \;\;\;\;\;\;\cdot \left(1+\frac{\left|\tau_{i}\left(s\right)\right|\cdot D_{h,i}\left(s\right)}{\kappa_{i}\left(s\right)+\epsilon }\right)+\gamma \cdot {\left(\frac{A_{ref}\left(s\right)-A_{i}\left(s\right)}{A_{ref}\left(s\right)}\right)}^{1.5} \end{array}$$

The geometric complexity indicator $w_{i}^{geo}\left(s\right)$ comprises three hemodynamically significant terms: The flow acceleration term (α-term) $\frac{\left|dA_{i}/ds\right|}{A_{i}\left(s\right)}\cdot \frac{Re_{i}\left(s\right)}{Re_{ref}}$ captures flow acceleration and deceleration effects through cross-sectional area variations weighted by local Reynolds number. This emphasizes regions where geometric transitions coincide with high-momentum flow conditions. The secondary flow term (β-term) incorporates curvature-induced secondary flow patterns through Dean flow analogy. The primary curvature effect $\frac{\kappa_{i}\left(s\right)\cdot D_{h,i}\left(s\right)}{2}\cdot \sqrt{\frac{Re_{i}\left(s\right)}{Re_{ref}}}$ is enhanced by the torsional factor $\left(1+\frac{\left|\tau_{i}\left(s\right)\right|\cdot D_{h,i}\left(s\right)}{\kappa_{i}\left(s\right)+\epsilon }\right)$, capturing three-dimensional helical flow patterns. The stenotic effect term (γ-term) ${\left(\frac{A_{ref}\left(s\right)-A_{i}\left(s\right)}{A_{ref}\left(s\right)}\right)}^{1.5}$ quantifies luminal narrowing relative to the reference area. The exponent 1.5 reflects the nonlinear relationship between area reduction and flow disturbance intensity.

The computed weights are normalized across all temporal phases as follows:

(8) $$w_{i}^{norm}\left(s\right)=w_{i}\left(s\right)/\sum_{j=0}^{10}w_{j}\left(s\right)$$

The final hemodynamically optimized geometric fusion is achieved through

(9) $$G_{avg}\left(s\right)=\sum_{i=0}^{10}w_{i}^{norm}\left(s\right)\times G_{i}\left(s\right)$$

where $G_{i}\left(s\right)$ represents the triangular mesh model of the coronary artery at arc length position s for temporal phase i. This weighted fusion process combines the mesh geometries from all cardiac phases to create a hemodynamically informed coronary artery model that reflects the flow environment experienced throughout the cardiac cycle.

This hemodynamically adjusted temporal fusion algorithm creates a flow-informed geometric representation that accounts for temporal flow variations and local vessel-specific hemodynamic characteristics. The fused geometry captures effective luminal dimensions encountered by blood flow over the cardiac cycle, with local adjustments reflecting frequency-dependent flow physics in different vessel segments. This approach addresses critical limitations of traditional static models, which may produce estimation errors for hemodynamic parameters such as FFR due to their inability to account for dynamic coronary geometry. By incorporating patient-specific cardiac mechanics and flow patterns, our temporal fusion method provides more physiologically representative boundary conditions for computational fluid dynamics analyses, potentially improving the clinical relevance of hemodynamic predictions and wall shear stress computations where accurate geometric representation directly influences velocity gradients at the vessel wall.

#### 2.2.3. Cardiac Function Quantitative Analysis

The accuracy of FFR_CT_ computation is directly dependent on the extraction of critical cardiac functional parameters from 4D-CTA data, which provide essential boundary conditions for coronary hemodynamic modeling. Our methodology focuses on quantifying core hemodynamic parameters that directly influence coronary blood flow distribution and pressure gradients, with particular emphasis on the precise determination of effective cardiac output.

Cardiac output represents the most critical parameter in FFR_CT_ modeling, as it determines the total blood flow entering the coronary arterial system. Through dynamic left ventricular models obtained from 4D CT imaging, we can precisely calculate left ventricular volume variations throughout the cardiac cycle. The left ventricular volume at time point $t_{i}$ is computed based on surface integration of the segmented endocardial boundary as follows:

(10) $$V_{LV}\left(t_{i}\right)={∭}_{\Omega_{LV}}dV=\frac{1}{3}\oint_{S_{LV}}\vec{r}\cdot \vec{n}dS$$

where $\vec{r}$ represents the position vector from an arbitrary origin to the surface element dS, and $\vec{n}$ denotes the unit normal vector to the surface. By identifying end-diastolic volume (EDV) and end-systolic volume (ESV) as the maximum and minimum values of the volume–time curve, the preliminary stroke volume is calculated as SV=EDV−ESV.

**Figure 5:**
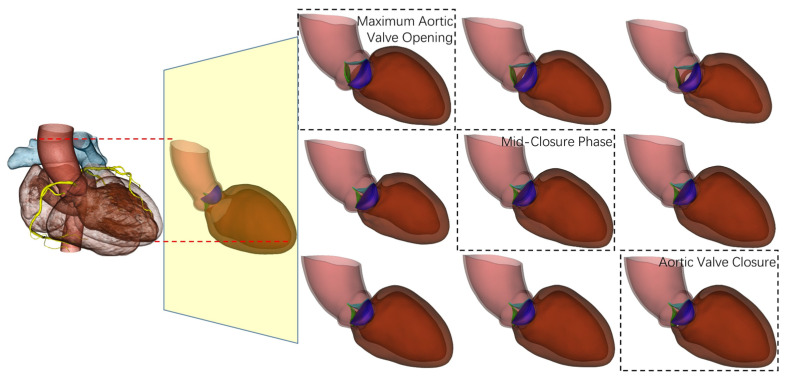
Dynamic representation of aortic valve function throughout the cardiac cycle.

However, stroke volume calculated solely from left ventricular volume changes does not fully represent the effective blood flow entering systemic circulation, as aortic regurgitation causes partial blood backflow to the left ventricle during diastole. The unique advantage of 4D-CTA lies in its capability to directly observe and quantify dynamic aortic valve behavior throughout the cardiac cycle (Figure 5), thereby enabling precise determination of regurgitant volume. As illustrated in Figure 5, the aortic valve demonstrates distinct phases from maximum opening during systole to complete closure during diastole, with intermediate mid-closure phases that can be accurately captured and analyzed using 4D-CTA imaging.

Aortic regurgitant volume is calculated through analysis of retrograde flow across the aortic valve region during diastole. Based on the dynamic aortic valve model reconstructed from 4D CT, regurgitant volume can be quantified as

(11) $$RV=\int_{t_{AVC}}^{t_{AVO}}Q_{retro}\left(t\right)dt$$

where $Q_{retro}\left(t\right)$ represents the retrograde flow through the aortic valve during diastole, and $t_{AVC}$ and $t_{AVO}$ denote aortic valve closure and opening times, respectively. In practical implementation, regurgitant volume can be estimated through analysis of diastolic cross-sectional area changes in the aortic root as follows:

(12) $$RV=\int_{z_{1}}^{z_{2}}\left[A_{aortic}\left(z,t_{dia}\right)-A_{aortic}\left(z,t_{AVC}\right)\right]dz$$

where $A_{aortic}\left(z,t\right)$ represents the aortic cross-sectional area at axial position z and time t, $z_{1}$ and $z_{2}$ denote the proximal and distal boundaries of the aortic root region, $t_{dia}$ represents mid-diastolic time when maximum regurgitation occurs, and $t_{AVC}$ is the time of aortic valve closure. The integration is performed over the entire aortic root region to capture the total regurgitant volume. Effective stroke volume is subsequently calculated as

(13) $${SV}_{eff}=SV-RV$$

This regurgitation-corrected calculation provides a more accurate representation of actual cardiac output, which is crucial for FFR_CT_ modeling, particularly in patients with aortic valve pathology.

Based on effective stroke volume, the true cardiac output is calculated as ${CO}_{eff}={SV}_{eff}\times HR$. In FFR_CT_ computation, this effective cardiac output is directly utilized to determine flow boundary conditions at the aortic root as follows:

(14) $$Q_{coronary}={CO}_{eff}\times f_{coronary}$$

where $f_{coronary}$ represents the fraction of effective cardiac output allocated to coronary circulation (typically 3–4% at rest [34]). This boundary condition setting based on effective cardiac output ensures that the FFR_CT_ model reflects the patient’s actual coronary perfusion status.

Aortic pressure parameters provide pressure boundary conditions for FFR_CT_ models, which are critical for accurate calculation of trans-stenotic pressure gradients. From 4D CT data, we estimate aortic pressure through analysis of ascending aortic geometric variations. The temporal changes in aortic cross-sectional area $A_{aortic}\left(t\right)$ exhibit direct correlation with pressure fluctuations, utilizing the Bramwell––Hill equation [35]

(15) $$\frac{\Delta P}{P_{0}}=\frac{1}{\beta }\frac{\Delta A_{aortic}}{A_{aortic,0}}$$

where $P_{0}$ and $A_{aortic,0}$ are reference pressure and area, ΔP and $\Delta A_{aortic}$ are their respective variations, and β is the arterial compliance coefficient. From this relationship, systolic and diastolic pressures ($P_{sys}$ and $P_{dia}$) are derived by analyzing the maximum and minimum pressure variations corresponding to peak aortic expansion and contraction phases. These pressures are then employed to establish outlet boundary conditions, typically using a three-element Windkessel model [36,37] as follows:

(16) $$P_{outlet}\left(t\right)=P_{sys}\exp\left(-t/\tau \right)+P_{dia}$$

where τ represents the arterial time constant, estimated from established cardiovascular models.

These cardiac functional parameters extracted from 4D-CTA are applied to FFR_CT_ computation through the following hierarchical approach: effective cardiac output determines coronary flow allocation, aortic pressure establishes pressure boundary conditions, and heart rate defines pulsatile frequency. In particular, the precise calculation of effective stroke volume, accounting for aortic regurgitation effects, ensures that FFR_CT_ models accurately reflect patients’ true hemodynamic status, thereby enhancing the clinical accuracy and reliability of virtual FFR measurements.

### 2.3. Enhanced FFR_CT_ Computational Framework

Based on the aforementioned temporally weighted dynamic geometric modeling and cardiac function quantitative analysis, we have established an enhanced FFR_CT_ computational framework that integrates dynamic geometric representation, physiology-guided boundary condition configuration, and optimized numerical solution strategies to improve the accuracy and clinical applicability of virtual FFR computations.

#### 2.3.1. Computational Fluid Dynamics Solver Configuration

We employ a customized solver based on the OpenFOAM platform for coronary artery hemodynamic computations [38]. Considering the pulsatile characteristics of coronary blood flow and complex geometric structures, the PIMPLE algorithm is selected as the core solution strategy, which combines the advantages of SIMPLE and PISO algorithms and effectively handles transient incompressible flow problems. The blood flow governing equations are based on the Navier–Stokes equations, assuming blood as an incompressible Newtonian fluid, with the continuity equation expressed as ∇⋅u=0 and the momentum equation as $\frac{\partial u}{\partial t}+\left(u\cdot \nabla \right)u=-\frac{1}{\rho }\nabla p+\frac{\mu }{\rho }\nabla^{2}u$, where u represents the velocity vector, p denotes pressure, $\rho =1060\mathrm{kg}/m^{3}$ is the blood density, and $\mu =3.71\times 10^{-3}\;\mathrm{Pa}\cdot s$ is the dynamic viscosity. Mesh generation employs an adaptive Cartesian mesh approach based on the cfMesh tool to achieve body-fitted mesh generation. To ensure accurate capture of boundary-layer flow, five layers of prismatic meshes are configured near vessel walls with the first layer height controlled at $y^{+}<1$ to satisfy the accuracy requirements for wall shear stress calculations.

#### 2.3.2. Dynamic Boundary Condition Configuration

**Figure 6:**
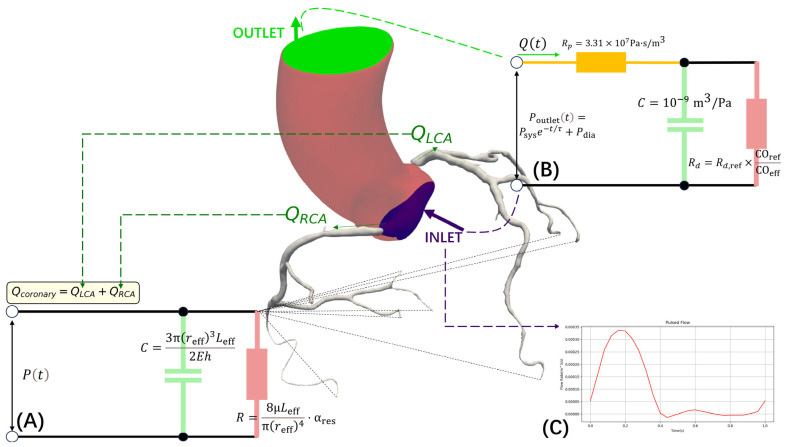
Computational domain decomposition and boundary condition implementation. (**A**) A 2-element Windkessel model of the outlet, (**B**) a 3-element Windkessel model for coronary branches, and (**C**) the pulsatile inlet flow waveform over a single cardiac cycle.

The aortic root inlet boundary condition employs a pulsatile velocity profile design based on effective cardiac output. Incorporating the effective cardiac output ${CO}_{eff}$ calculated in Section 2.2.3, the inlet flow waveform is defined as

(17) $$Q_{inlet}\left(t\right)={CO}_{eff}\cdot f_{temporal}\left(t\right)$$

where $f_{temporal}\left(t\right)$ represents a normalized cardiac cycle flow function fitted based on physiological data as $f_{temporal}\left(t\right)=A_{1}\sin\left(\omega t+\phi_{1}\right)+A_{2}\sin\left(2\omega t+\phi_{2}\right)+A_{3}$, with parameters $A_{1}=0.8$, $A_{2}=0.3$, $A_{3}=0.1$, $\phi_{1}=\pi /4$, and $\phi_{2}=\pi /6$ calibrated according to typical aortic flow waveforms [39]. As illustrated in Figure 6C, the inlet flow waveform exhibits the characteristic pulsatile pattern over a single cardiac cycle, capturing the physiological variations in aortic flow. The velocity profile adopts the Womersley analytical solution, considering the frequency-dependent characteristics of pulsatile flow, expressed as $u_{z}\left(r,t\right)=\frac{Q_{inlet}\left(t\right)}{\pi R^{2}}\cdot \left[1-\frac{J_{0}\left(\alpha_{n}r/R\right)}{J_{0}\left(\alpha_{n}\right)}\right]$, where r represents the radial distance from the vessel centerline, R denotes the vessel radius, $J_{0}$ is the zero-order Bessel function, and $\alpha_{n}$ represents the complex roots of the Womersley number [40].

The boundary condition implementation strategy is comprehensively depicted in Figure 6, which shows the computational domain decomposition with different models applied at various outlets. The aortic outlet employs a three-element Windkessel model to characterize the complex impedance characteristics of systemic circulation (Figure 6B) as follows:

(18) $$P\left(t\right)=R_{p}\cdot Q\left(t\right)+P_{d}\left(t\right)$$

(19) $$\frac{dP_{d}}{dt}=\frac{Q\left(t\right)-P_{d}\left(t\right)/R_{d}}{C}$$

where $P_{d}\left(t\right)$ represents the diastolic pressure component across the distal resistance, and the characteristic impedance $R_{p}=3.31\times 10^{7}$ $\mathrm{Pa}\cdot s/m^{3}$ and arterial compliance $C=1.0\times 10^{-9}$ $m^{3}/\mathrm{Pa}$ are set to standard physiological values [15]. The peripheral resistance is adjusted based on the effective cardiac output calculated in Section 2.2.3 as follows: $R_{d}=R_{d,ref}\times \frac{{CO}_{ref}}{{CO}_{eff}}$, where $R_{d,ref}=3.35\times 10^{8}$ $\mathrm{Pa}\cdot s/m^{3}$ represents the reference peripheral resistance and ${CO}_{ref}=5.0$ L/min is the reference cardiac output.

Coronary branch outlets employ a two-element Windkessel model incorporating patient-specific parameters for boundary condition configuration (Figure 6A). This model describes the pressure–flow relationship at outlets through the differential equation $R\frac{dQ}{dt}+\frac{Q}{C}=\frac{dP}{dt}$, where Windkessel parameters are automatically calculated based on patient-specific vessel geometry and cardiac functional parameters. The peripheral resistance $R=\frac{8\mu L_{eff}}{\pi {\left(r_{eff}\right)}^{4}}\cdot \alpha_{res}$ is calculated based on effective vessel parameters, where $r_{eff}$ represents the temporally weighted average radius obtained through $r_{eff}\left(s\right)=\sum_{i=0}^{10}w_{i}^{norm}\left(s\right)\times r_{i}\left(s\right)$ (as defined in Section 2.2.2), and $L_{eff}$ denotes the effective vessel length calculated as the integrated centerline length from the outlet to the main coronary ostium based on the fused geometric model $G_{avg}\left(s\right)$. The resistance correction factor $\alpha_{res}$ is determined by the ratio of individual myocardial perfusion territory volume (segmented from patient CT imaging) to the reference perfusion volume. The compliance $C=\frac{3\pi {\left(r_{eff}\right)}^{3}L_{eff}}{2Eh}$ reflects the elastic properties of vessel walls, where the elastic modulus E is estimated based on patient age and cardiovascular risk factors, and wall thickness h is derived from vessel geometry analysis [41,42]. This differentiated boundary condition approach, as demonstrated in Figure 6, ensures that each outlet type is modeled with appropriate complexity to accurately capture the distinct hemodynamic characteristics of systemic and coronary circulations. This patient-specific parameterization ensures that outlet boundary conditions accurately represent individual hemodynamic characteristics and myocardial perfusion demands.

#### 2.3.3. CFD Implementation of Temporally Weighted Geometry

**Figure 7:**
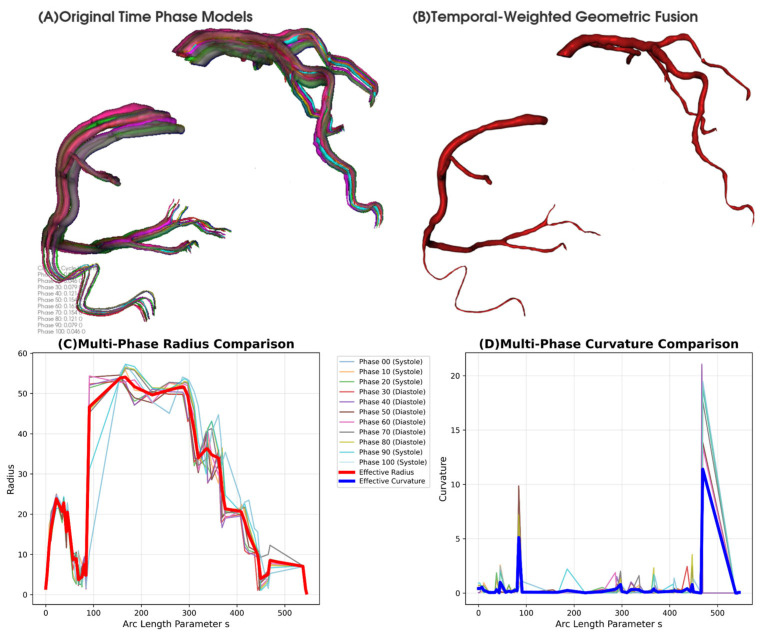
Temporally weighted geometric fusion of coronary artery models. (**A**) Original time phase models across cardiac cycle (phases 0–100). (**B**) Temporally weighted fusion result. (**C**) Multi-phase radius comparison with effective radius (red line). (**D**) Multi-phase curvature comparison with effective curvature (blue line). Systolic (0–20, 90–100) and diastolic phases (30–80) shown with different line styles.

The temporally weighted fused geometry $G_{avg}\left(s\right)$ obtained from Section 2.2.2 is implemented in CFD calculations through geometric interpolation strategies that realize the spatial distribution of fused geometric parameters at CFD mesh nodes. The temporally weighted geometric fusion process is illustrated in Figure 7, where the original time phase models (Figure 7A) demonstrate dynamic vessel geometry variations across the cardiac cycle with different colors representing phases 0–100%. While $G_{avg}\left(s\right)$ represents the complete fused triangular mesh model, CFD simulations require specific geometric parameters for numerical computations. The temporally weighted geometric fusion result showing the averaged geometry is presented in Figure 7B. Therefore, key geometric parameters are extracted from the temporal fusion process: the effective radius $r_{eff}\left(s\right)=\sum_{i=0}^{10}w_{i}^{norm}\left(s\right)\times r_{i}\left(s\right)$ and effective curvature $\kappa_{eff}\left(s\right)=\sum_{i=0}^{10}w_{i}^{norm}\left(s\right)\times \kappa_{i}\left(s\right)$. Multi-phase radius comparison along arc length parameter s is shown in Figure 7C, while multi-phase curvature comparison is presented in Figure 7D. These parameters are calculated using the same temporal weighting scheme to ensure consistency with the fused geometry. In CFD calculations, $r_{eff}\left(s\right)$ determines the local cross-sectional area affecting velocity profiles and pressure gradients, while $\kappa_{eff}\left(s\right)$ influences wall shear stress distribution and secondary flow patterns in curved vessel segments. This approach ensures that geometric parameters used in CFD calculations reflect the effective resistance characteristics encountered by blood flow throughout the cardiac cycle. Standard CFD numerical methods are employed to ensure solution stability when implementing the temporally weighted geometric parameters [43].

#### 2.3.4. Enhanced FFR Computation Framework

FFR values are computed as the pressure ratio $FFR=P_{d}/P_{a}$, where $P_{d}$ represents the distal stenotic pressure and $P_{a}$ denotes aortic pressure. Considering the influence of pulsatile flow, time-averaged pressure $\bar{P}=\frac{1}{T}\int_{0}^{T}P\left(t\right)dt$ is employed for FFR calculations to ensure clinical relevance of results. The core advantage of this enhanced framework lies in the accurate capture of dynamic geometric effects, where temporally weighted geometry can reflect the effective resistance characteristics of vessels throughout the cardiac cycle compared to traditional static geometric methods, particularly for complex flow phenomena in curved and bifurcated regions. By configuring boundary conditions through effective cardiac output ${CO}_{eff}$, the framework avoids flow overestimation issues caused by neglecting aortic regurgitation in traditional methods, while patient-specific automatic generation of Windkessel parameters further enhances individualized computational accuracy.

This enhanced FFR_CT_ computational framework integrates dynamic geometric modeling, physiology-guided boundary conditions, and optimized numerical methods to improve the accuracy of clinical FFR assessment. The temporally weighted geometry approach captures dynamic vessel characteristics throughout the cardiac cycle, while the effective cardiac output-based boundary conditions account for individual physiological variations. The framework is particularly applicable to functional evaluation of complex coronary artery lesions with geometric variations.

## 3. Results

To demonstrate the clinical feasibility and accuracy of our proposed 4D-CTA based FFR_CT_ methodology, we present a representative case with invasive FFR measurements serving as the reference standard. This proof-of-concept analysis compares our novel 4D dynamic approach against both invasive FFR and conventional static FFR_CT_ calculations. The conventional method utilizes single-phase end-diastolic CTA data, while our 4D approach incorporates temporal coronary dynamics throughout the complete cardiac cycle. This case demonstrates the potential advantages of dynamic 4D-CTA data in capturing physiologically relevant hemodynamic parameters for non-invasive functional coronary assessment, providing initial evidence for the clinical utility of this innovative computational approach.

### 3.1. Invasive FFR Assessment

**Figure 8:**
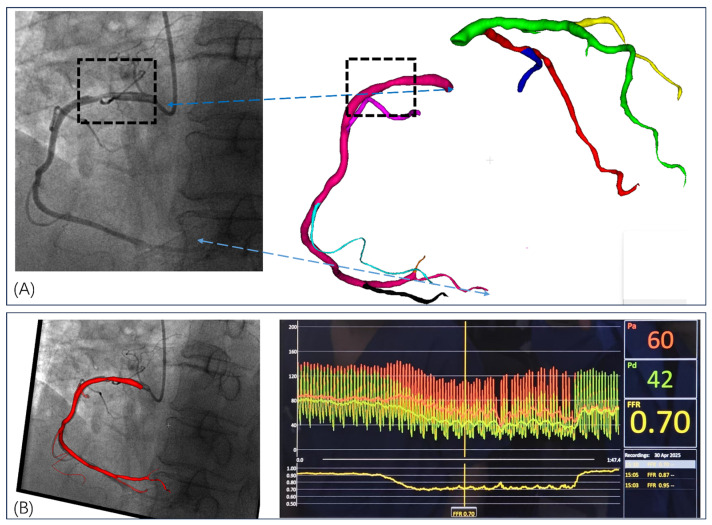
Invasive FFR Assessment of Right Coronary Artery Stenosis. (**A**) Coronary angiography showing significant stenosis in the proximal right coronary artery (dashed box) with corresponding 3D coronary tree reconstruction from 4D-CTA data. Color-coded segments represent different coronary branches used for computational analysis. (**B**) Real-time invasive FFR measurement displaying simultaneous aortic pressure (Pd: 60 mmHg) and distal coronary pressure (Pa: 42 mmHg) recordings during adenosine-induced hyperemia, yielding an FFR value of 0.70, indicating hemodynamically significant stenosis.

Invasive FFR was measured during cardiac catheterization using a 0.014-inch pressure-sensitive guidewire positioned distal to the proximal right coronary artery stenosis (Figure 8). Maximal hyperemia was induced with intravenous adenosine infusion (140 μg/kg/min), and simultaneous aortic and distal coronary pressures were recorded continuously. As demonstrated in Figure 8B, the real-time pressure tracings showed stable hyperemic conditions with aortic pressure of 60 mmHg and distal coronary pressure of 42 mmHg. FFR was calculated as the ratio of mean distal coronary pressure to mean aortic pressure ($P_{d}/P_{a}$) during steady-state hyperemia, averaged over multiple cardiac cycles. The invasive FFR measurement was 0.70, indicating hemodynamically significant stenosis requiring revascularization (threshold < 0.80). The corresponding angiographic images and 3D coronary reconstruction (Figure 8A) provided anatomical correlation for the stenotic segment used in subsequent computational FFR_CT_ analysis. Hemodynamic parameters including blood pressure, heart rate, and cardiac rhythm were continuously monitored throughout the procedure to ensure measurement accuracy and patient safety. This invasive measurement served as the reference standard for validation of computational FFR_CT_ derived from the corresponding 4D-CTA dataset.

### 3.2. FFR_CT_ Computational Results

**Figure 9:**
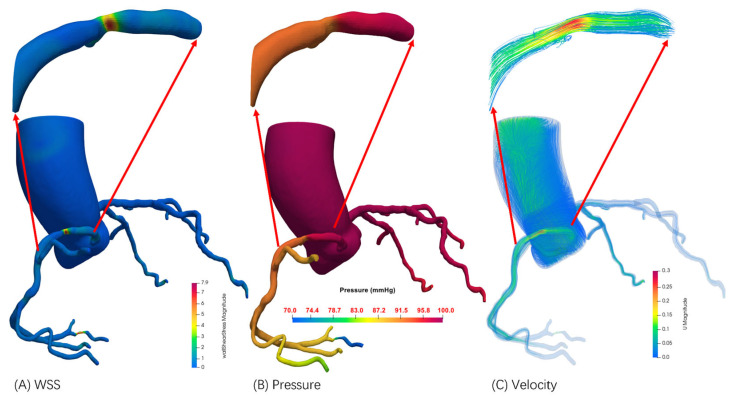
Steady-state hemodynamic distributions in the coronary artery model. The visualization displays (**A**) wall shear stress (WSS) magnitude, (**B**) pressure field, and (**C**) velocity magnitude throughout the computational domain.

Computational FFR was derived from the patient-specific 4D-CTA dataset using our enhanced time-weighted geometric fusion framework and personalized hemodynamic modeling approach (Figure 9). The computational domain was reconstructed from the same coronary anatomy evaluated invasively, incorporating patient-specific geometric features extracted from temporal CTA phases to capture dynamic vessel characteristics throughout the cardiac cycle.

Patient-specific computational parameters were individualized based on clinical data and 4D-CTA measurements. The computational mesh comprised 151,228 surface elements with adaptive refinement applied to patient-specific bifurcation regions and areas of geometric complexity identified from the 4D-CTA reconstruction. Boundary layer meshing was customized according to individual vessel dimensions, with layer thickness optimized for accurate near-wall hemodynamic computation in this patient’s coronary geometry. Blood rheological properties were personalized based on patient demographics and hematological parameters obtained during clinical evaluation.

Inlet boundary conditions were derived from patient-specific pulsatile flow waveforms extracted from the 4D-CTA temporal analysis, reflecting individual cardiac cycle characteristics and flow patterns. Outlet impedance values were calculated from patient-specific cardiac output and peripheral resistance measurements, ensuring physiologically realistic boundary conditions tailored to this patient’s cardiovascular profile. The simulation was performed over three complete cardiac cycles at the patient’s recorded heart rate to achieve periodic steady-state conditions.

Figure 9 demonstrates the computed hemodynamic distribution within the patient-specific coronary model. The results reveal complex flow patterns characteristic of this patient’s coronary circulation, with significant spatial variations in hemodynamic parameters corresponding to the individual vessel geometry and stenotic regions identified in the invasive evaluation. WSS distribution (Figure 9A) exhibited marked spatial heterogeneity reflecting the patient’s unique vascular geometry, with stress concentrations observed in bifurcation regions and the stenotic segment corresponding to the invasively evaluated lesion. The pressure field (Figure 9B) demonstrated the characteristic pressure drop across the stenotic region, with computational values showing excellent correlation with invasively measured pressures. Velocity magnitude distribution (Figure 9C) captured acceleration and deceleration patterns specific to this patient’s vessel geometry, with peak velocities observed in the stenotic segment consistent with angiographic findings.

**Figure 10:**
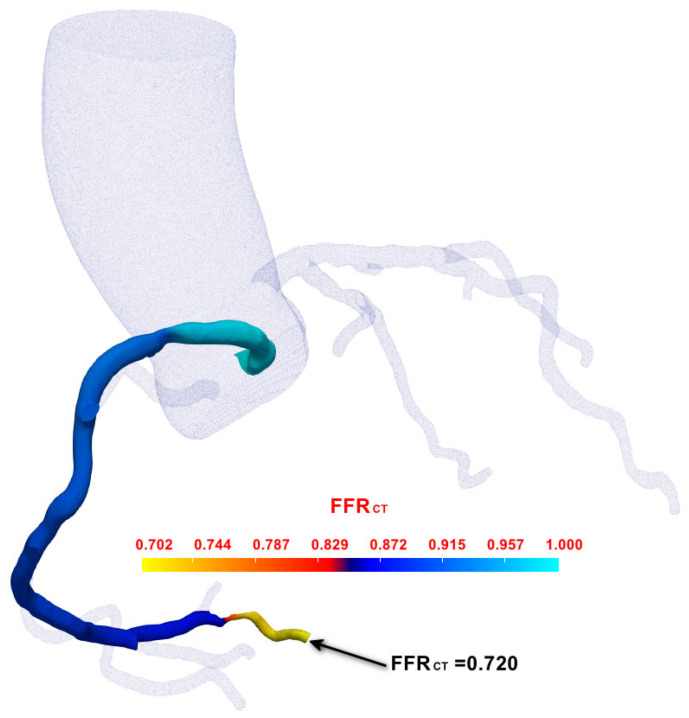
4D-CTA-based FFR_CT_ computation results for the RCA.

Following comprehensive hemodynamic analysis, we applied our novel 4D-CTA-based FFR_CT_ computation method to functionally assess the RCA. Unlike conventional static CTA-based approaches, our 4D-CTA method captures dynamic vessel geometry changes and time-varying flow characteristics throughout the cardiac cycle. As illustrated in Figure 10, the computed FFR_CT_ distribution along the RCA demonstrates progressive pressure reduction from ostium to distal segments, with color-coded visualization revealing spatial FFR_CT_ variations that account for cardiac cycle dynamics. The computed FFR_CT_ value of 0.720 at the critical stenotic region closely approximated the invasively measured FFR value of 0.70 (difference of 0.02), providing initial validation of the feasibility of our 4D-CTA-based dynamic modeling approach. Our method successfully captured the influence of vessel wall motion and pulsatile flow on pressure distribution throughout the cardiac cycle, demonstrating the technical feasibility and clinical potential of 4D-CTA-based dynamic FFR_CT_ computation for non-invasive coronary functional assessment.

### 3.3. Comparative Validation Results

To comprehensively evaluate the accuracy and clinical utility of our 4D-CTA-based dynamic FFR_CT_ methodology, we conducted comparative analysis across multiple patient cases with invasive FFR measurements as the reference standard. Three patient cases were analyzed, including one case with dual-vessel FFR measurements, providing a total of four coronary vessel assessments for validation.

#### 3.3.1. Patient Demographics and Clinical Characteristics

The validation cohort comprised three patients (aged 72, 61, and 56 years, respectively) with suspected coronary artery disease who underwent both invasive coronary angiography with FFR measurements and 4D-CTA imaging within approximately one week. All patients provided informed consent, and the study protocol was approved by the institutional review board. Clinical characteristics included stable angina presentation, preserved left ventricular function, and the absence of acute coronary syndromes.

#### 3.3.2. Computational Methodologies

Two computational approaches were compared against invasive FFR:

Conventional Static FFR_CT_: Following established protocols from the CoronaryHemodynamics framework [38], computational domains were reconstructed from end-diastolic-phase CTA data. Patient-specific boundary conditions included Windkessel outlet parameters automatically derived from physiological metrics (heart rate, systolic blood pressure, and myocardial volume), with parabolic inlet flow profiles applied based on empirical flow rate formulas. Steady-state simulations were performed using the SIMPLE algorithm with standard OpenFOAM solvers, incorporating body-fitted cartesian mesh generation via cfMesh with adaptive refinement at bifurcation regions.

Our 4D-CTA Dynamic FFR_CT_: Our novel approach utilized temporal geometric fusion across multiple cardiac phases, incorporating time-varying vessel geometry and pulsatile flow characteristics. Patient-specific hemodynamic parameters were individualized based on 4D-CTA temporal analysis, with personalized inlet boundary conditions derived from cardiac cycle-specific flow waveforms and outlet impedance values calculated from patient-specific cardiac output measurements.

#### 3.3.3. Validation Results

**Figure A1:**
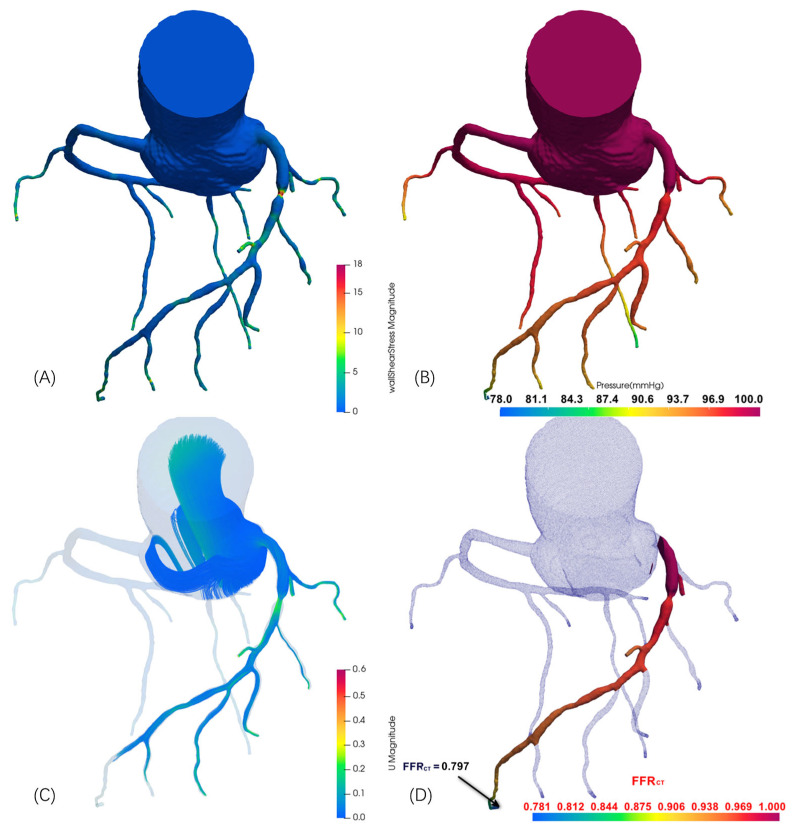
Steady-state hemodynamic distributions in the coronary artery model for Case 2. The visualization displays (**A**) Wall Shear Stress (WSS) magnitude, (**B**) pressure field, (**C**) velocity magnitude, and (**D**) FFR_CT_ computation results throughout the computational domain.

**Figure A2:**
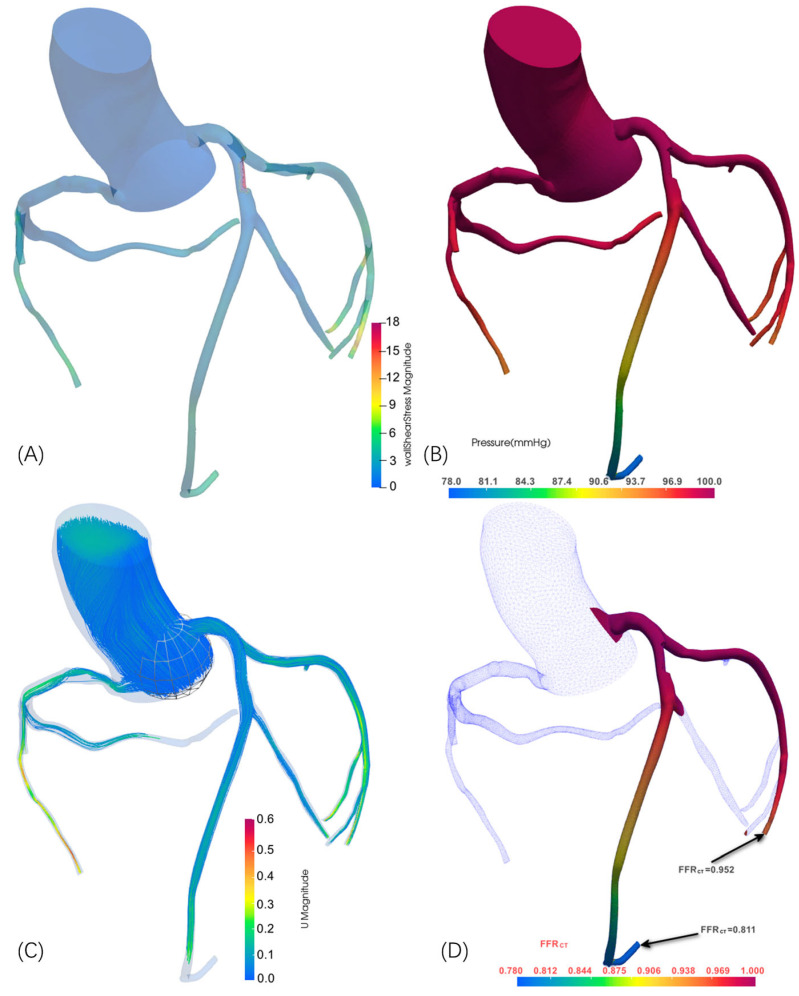
Steady-state hemodynamic distributions in the coronary artery model for Case 3. The visualization displays (**A**) Wall Shear Stress (WSS) magnitude, (**B**) pressure field, (**C**) velocity magnitude, and (**D**) FFR_CT_ computation results throughout the computational domain.

The preliminary validation results demonstrate the technical feasibility of our 4D-CTA based dynamic FFR_CT_ computation approach. Across all four vessel assessments, the dynamic method successfully generated FFR_CT_ values that closely approximated invasive FFR measurements, with individual errors ranging from 0.008 to 0.033. Compared to static FFR_CT_ (errors: 0.021–0.045), the dynamic approach showed improved accuracy, particularly in the diagnostically critical 0.75–0.85 FFR range (Cases 2 and 3a) where measurement precision has the greatest impact on clinical decision-making. The dynamic approach effectively incorporated temporal vessel geometry variations and cardiac cycle-specific flow characteristics, demonstrating its capability to capture physiologically relevant hemodynamic parameters that static methods may overlook. Case 1 results are illustrated in Figure 9 and Figure 10, while Case 2 and Case 3 results are presented in Figure A1 and Figure A2, respectively.

Table 1 presents the preliminary validation results from our limited cohort of 3 patients. While the visual correlation appears promising, these results should be interpreted with caution given the small sample size. While the limited sample size precludes definitive conclusions about comparative performance, these initial results provide encouraging evidence for the clinical potential of 4D-CTA based FFR_CT_ computation. The dynamic method successfully processed complex temporal imaging data and generated physiologically plausible hemodynamic assessments across diverse coronary anatomical configurations. These findings support the technical feasibility of implementing 4D-CTA temporal information for non-invasive functional coronary assessment, warranting further investigation in larger patient cohorts to establish comprehensive validation and clinical utility.

## 4. Discussion

This study presents a novel computational framework for dynamic FFR_CT_ calculation using 4D-CTA imaging data, demonstrating the technical feasibility of incorporating temporal cardiac dynamics into virtual FFR assessment. The methodology successfully integrates advanced image processing, patient-specific geometric modeling, and efficient computational algorithms to provide dynamic hemodynamic evaluation throughout the cardiac cycle.

### 4.1. Technical Innovation and Clinical Significance

The primary innovation of this work lies in the transition from static to dynamic FFR_CT_ computation, addressing a significant limitation in current computational approaches. Traditional FFR_CT_ methods rely on single-phase imaging and static boundary conditions, potentially missing critical hemodynamic variations that occur during the cardiac cycle, including systolic acceleration, diastolic deceleration, and flow reversal patterns. Our 4D-CTA based approach captures these temporal variations, providing a more physiologically realistic assessment of coronary stenosis severity throughout the cardiac cycle.

Our framework reveals dynamic hemodynamic behaviors that are not captured by traditional static approaches but may influence stenosis functional significance. The ability to incorporate time-varying geometries and flow patterns may contribute to more personalized cardiovascular assessment, potentially addressing important limitations of the standardized approaches commonly used in current computational methodologies.

### 4.2. Methodological Advantages

The dynamic boundary condition implementation based on 4D-CTA flow measurements offers an alternative to empirically derived parameters commonly used in existing methods. This patient-specific approach may help address some aspects of inter-patient variability that can affect diagnostic accuracy in standardized computational frameworks. By incorporating actual temporal flow patterns rather than assumed waveforms, our methodology potentially provides more individualized hemodynamic assessment based on each patient’s cardiac dynamics.

The temporal resolution of 4D-CTA allows capture of pulsatile flow dynamics and vessel wall motion, phenomena that may influence coronary hemodynamics but are typically not considered in static analyses [24]. This capability could be particularly relevant for assessing functional significance of intermediate stenoses, where dynamic flow patterns might provide additional hemodynamic information not captured in static evaluations.

Our framework represents a step toward more individualized hemodynamic evaluation, moving beyond some of the standardized computational assumptions applied uniformly across patients.

### 4.3. Study Limitations and Future Directions

The current validation is based on a limited dataset, reflecting the exploratory nature of this technical feasibility study. While the small sample size limits statistical power and generalizability, it provides essential proof-of-concept evidence for the proposed methodology. The primary objective at this stage is to demonstrate technical feasibility and establish the computational framework rather than comprehensive clinical validation.

Future work will focus on expanding the validation cohort to include diverse patient populations and stenosis severities. Large-scale clinical studies comparing dynamic FFR_CT_ against invasive FFR measurements will be essential for establishing diagnostic accuracy and clinical utility. Additionally, optimization of computational algorithms to reduce processing time will be crucial for real-time clinical implementation.

The current framework assumes rigid vessel walls, which may not fully capture the complex biomechanical interactions in diseased coronary arteries. Future developments should incorporate fluid–structure interaction modeling to account for vessel compliance and wall motion effects on hemodynamic patterns.

### 4.4. Clinical Implementation Considerations

The integration of dynamic FFRCT into routine clinical practice will require consideration of workflow feasibility and implementation challenges. The methodology could be incorporated into existing cardiac CT protocols with minimal additional patient burden, as 4D-CTA acquisition can be performed during routine coronary CTA examinations.

Key implementation requirements include standardized acquisition protocols, offline computational processing capabilities, and integration with existing imaging systems. The approach may be particularly valuable for intermediate stenosis cases where conventional imaging provides ambiguous results, potentially reducing unnecessary invasive procedures.

Future clinical adoption will depend on validation studies demonstrating improved diagnostic accuracy and cost-effectiveness compared to current standard-of-care approaches, as well as optimization of processing times for practical clinical workflows.

## 5. Conclusions

This study demonstrates the technical feasibility of dynamic FFR_CT_ computation using 4D-CTA imaging data. The developed computational framework integrates temporal cardiac dynamics into virtual FFR evaluation, potentially addressing some limitations of existing static methodologies.

Key achievements include implementation of a 4D-CTA to CFD pipeline and development of dynamic boundary conditions based on patient-specific flow measurements. The methodology’s ability to capture temporal hemodynamic variations throughout the cardiac cycle may offer improved diagnostic capabilities for coronary artery disease assessment.

The clinical implications are significant: enhanced diagnostic accuracy could improve patient selection for revascularization procedures, reduce unnecessary invasive catheterizations, and potentially decrease healthcare costs while maintaining patient safety. This non-invasive approach may particularly benefit intermediate-risk patients where treatment decisions are challenging.

While current validation is limited, reflecting the exploratory nature of this feasibility study, the results establish a foundation for dynamic coronary hemodynamic assessment. Future research priorities include validation in large multicenter cohorts, comparison with invasive FFR measurements, and development of real-time computational capabilities for clinical integration.

This work contributes to the development of more physiologically relevant non-invasive FFR evaluation methods, with potential implications for improved coronary artery disease diagnosis and establishing the groundwork for next-generation dynamic cardiac imaging applications.

## Figures and Tables

**Table 1:** Summarizes the comparative FFR_CT_ results across all four vessel assessments.

Case	Vessel	Invasive FFR	Static FFR_CT_	Dynamic FFR_CT_
1	RCA	0.70	0.742	0.720
2	LAD	0.78	0.825	0.797
3a	LAD	0.78	0.818	0.811
3b	LCX	0.94	0.961	0.952

## Data Availability

The original contributions presented in this study are included in the article. Further inquiries can be directed to the corresponding author.

## Abbreviations

The following abbreviations are used in this manuscript:

4D-CTA	Four-dimensional computed tomography angiography
CAD	Coronary artery disease
CCTA	Coronary computed tomography angiography
CFD	Computational Fluid Dynamics
CVDs	Cardiovascular diseases
EDV	End-diastolic volume
ESV	End-systolic volume
FFR	Fractional Flow Reserve
FFRCT	FFR derived from CCTA
QFR	Quantitative Flow Ratio
vFFR	Virtual FFR
